# Lean and Obese Zucker Rat Extensor Digitorum Longus Muscle high-frequency electrical stimulation (HFES) Data: Regulation of p70S6kinase Associated Proteins

**DOI:** 10.1016/j.dib.2017.11.051

**Published:** 2017-11-20

**Authors:** Kevin M. Rice, Anjaiah Katta, Nandini D.P.K. Manne, Ravikumar Arvapalli, Gautam K. Ginjupalli, Miaozong Wu, Shinichi Asano, Eric R. Blough

**Affiliations:** a Center for Diagnostic Nanosystems, Marshall University, Huntington, WV, USA; b Department of Internal Medicine, Joan C. Edwards School of Medicine, Marshall University, Huntington, WV, USA; c Biotechnology Graduate Program West Virginia State University, Institute, WV, USA; d Department of Health and Human Service, School of Kinesiology, Marshall University, Huntington, WV, USA; e Department of Public Heath, Marshall University, Huntington, WV, USA; f College of Health, Science, and Technology, University of Central Missouri, Warrensburg, MO, USA; g School of Education, Health, and Human Performance, Fairmont State University, Fairmont, WV, USA; h Department of Pharmaceutical Sciences and Research, School of Pharmacy, Marshall University, Huntington, WV, USA; i Department of Pharmacology, Physiology and Toxicology, Joan C. Edwards School of Medicine, Marshall University, Huntington, WV, USA

**Keywords:** Diabetes, Skeletal muscle, High-frequency electrical stimulation (HFES), Zucker rat, Extensor Digitorum Longus, p70s6k

## Abstract

Anaerobic exercise has been advocated as a prescribed treatment for the management of diabetes: however, alterations in exercise-induced signaling remain largely unexplored in the diabetic muscle. Here, we compare the basal and the in situ contraction-induced phosphorylation of the AKT, GSK3beta, mTor, p70s6K, Pten, and Shp2 in the lean and obese (fa/fa) Zucker rat Extensor Digitorum Longus (EDL) muscle following a single bout of contractile stimuli. This article represents data associated with prior publications from our lab (Katta et al., 2009a, 2009b; Tullgren et al., 1991) [1–3] and concurrent Data in Brief articles (Ginjupalli et al., 2017a, 2017b; Rice et al., 2017a, 2017b) [4–7].

**Specifications Table**

**Table:** 

Subject area	*Biology*
More specific subject area	*Diabetic skeletal muscle response to exercise*
Type of data	*Graph, figure*
How data was acquired	*Immunoblotting*
Data format	*Analyzed*
Experimental factors	*A high-frequency electrical stimulation (HFES) was used to produce 10 sets of 6 contractions over a 22-min period. Tissues were collected and protein was then isolated from tissue for western blot analysis.*
Experimental features	*EDL obtained from Lean and Obese male Zucker rats were used in this experiment*
Data source location	*Huntington, WV USA*
Data accessibility	*Data is with this article and is related to articles published and in review*[1], [2], [3], [4], [5], [6], [7]

**Value of the data**

• The data presented in this Brief is vital to understanding the effect of diabetes on skeletal muscle mechanotransduction.
• This data gives insight into the how diabetes alters tissue response to stimuli.
• This data provides a more thorough understanding of the mTor pathway involvement in exercise mediated signaling in both diabetic and non-diabetic muscle tissue.

## 1 Data

### 1.1 AKT

**Fig. 1:**
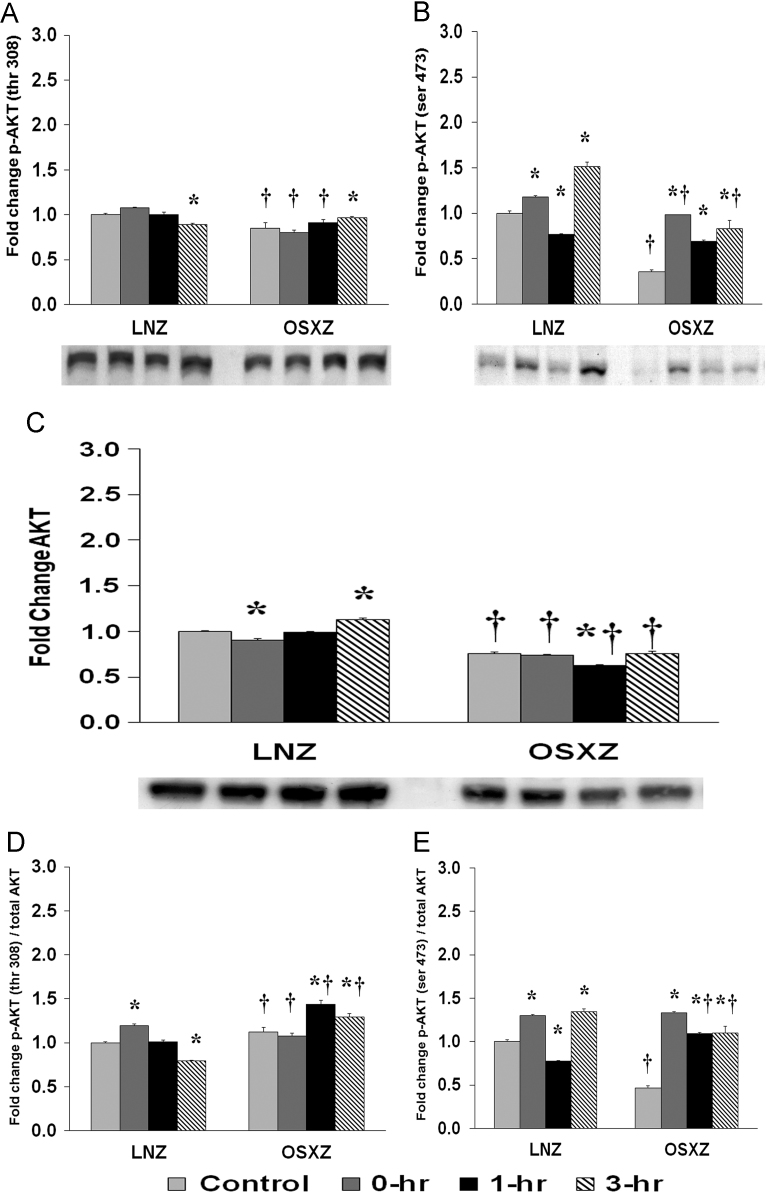
Diabetes alters HFES-induced expression and phosphorylation of Akt rat EDL. The basal (control) and HFES-induced expression of Akt in EDL from non-diabetic lean Zucker (LNZ) and diabetic obese syndrome X Zucker (OSXZ) rats. * Significantly different from HFES EDL within the same group (*p* < 0.05). † Significantly different from corresponding LNZ EDL (*p* < 0.05). *n* = 6/group.

To determine the effect of HFES on EDL from diabetic male obese syndrome-X Zucker (OSXZ) diabetic and nondiabetic male normal lean Zucker (LNZ) animals we evaluated the phosphorylation of AKT at Threonine 308 and Serine 473. EDL basal phosphorylation of AKT Thr 308 was lower (15.0 ± 5.9%, *p* < 0.05) in the OSXZ when compared to LNZ (Fig. 1A). HFES resulted in a decrease in phosphorylation of AKT Thr 308 in the LNZ EDL (10.9 ± 1.6% at 3 h, *p* < 0.05) when compared to LNZ contralateral control (Fig. 1A). HFES resulted in an increase in phosphorylation of AKT Thr 308 in the OSXZ EDL (11.9 ± 1.5%, at 3 h, *p* < 0.05) when compared to OSXZ contralateral control (Fig. 1A). EDL basal phosphorylation of AKT Ser 473 was lower (64.6 ± 2.6%, *p* < 0.05) in the OSXZ when compared to LNZ (Fig. 1B). HFES resulted in an increase in phosphorylation of AKT Ser 473 in the LNZ EDL (17.8 ± 1.6%, and 51.5 ± 4.9%, at 0 and 3 h, *p* < 0.05) and decrease (23.2 ± 1.0%, 1 h, *p* < 0.05) when compared to LNZ contralateral control (Fig. 1B). HFES resulted in an increase in phosphorylation of AKT Ser 473 in the OSXZ EDL (63.0 ± 0.3%, 33.7 ± 1.6%, and 47.9 ± 8.7%, at 0, 1 and 3 h, *p* < 0.05) when compared to OSXZ contralateral control (Fig. 1B).

To determine the effect of HFES on EDL from OSXZ and LNZ animals we evaluated the expression of AKT. EDL basal AKT content was lower (24.4 ± 1.7%, *p* < 0.05) in the OSXZ when compared to LNZ (Fig. 1C). HFES resulted in a decrease in AKT in the LNZ EDL (9.3 ± 1.7%, at 0 h, *p* < 0.05) and increase (12.5 ± 1.9%, at 3 h, *p* < 0.05) when compared to LNZ contralateral control (Fig. 1C). However HFES resulted in a decrease in AKT (12.5 ± 0.8%, at 1 h, *p* < 0.05) in the OSZX EDL when compared to contralateral OXSZ control (Fig. 1C).

To determine the effect of HFES on EDL from OSXZ and LNZ animals we evaluated the phosphorylation of AKT Thr 308 and AKT Ser 473 to total AKT. EDL basal phosphorylation of AKT Thr 308 to total AKT was higher (12.1 ± 5.6%, *p* < 0.05) in the OSXZ when compared to LNZ (Fig. 1D). HFES resulted in an increase in phosphorylation of AKT Thr 308 to total AKT in the LNZ EDL (19.3 ± 2.0%, at 0 h, p < 0.05) and lower (20.8 ± 0.5%, at 3 h, p < 0.05) when compared to LNZ contralateral control (Fig. 1D). HFES resulted in an increase in phosphorylation of AKT Thr 308 to total AKT in the OSXZ EDL (31.9 ± 4.4% and 17.3 ± 4.1%, at 0 and 3 hours, *p* < 0.05) when compared to OSXZ contralateral control (Fig. 1D). EDL basal phosphorylation of AKT Ser 473 to total AKT was lower (53.4 ± 2.4%, *p* < 0.05) in the OSXZ when compared to LNZ (Fig. 1E). HFES resulted in an increase in phosphorylation of AKT Ser 473 to total AKT in the LNZ EDL (30.1 ± 1.4%, and 34.6 ± 2.9%, at 0 and 3 h, *p* < 0.05) and lower (22.6 ± 0.7%, at 1 h, *p* < 0.05) when compared to LNZ contralateral control (Fig. 1E). HFES resulted in an increase in phosphorylation of AKT Ser 473 to total AKT in the OSXZ EDL (222.4 ± 36.7% at 0 h, *p* < 0.05) when compared to OSXZ contralateral control (Fig. 1E).

### 1.2 GS3K-β

**Fig. 2:**
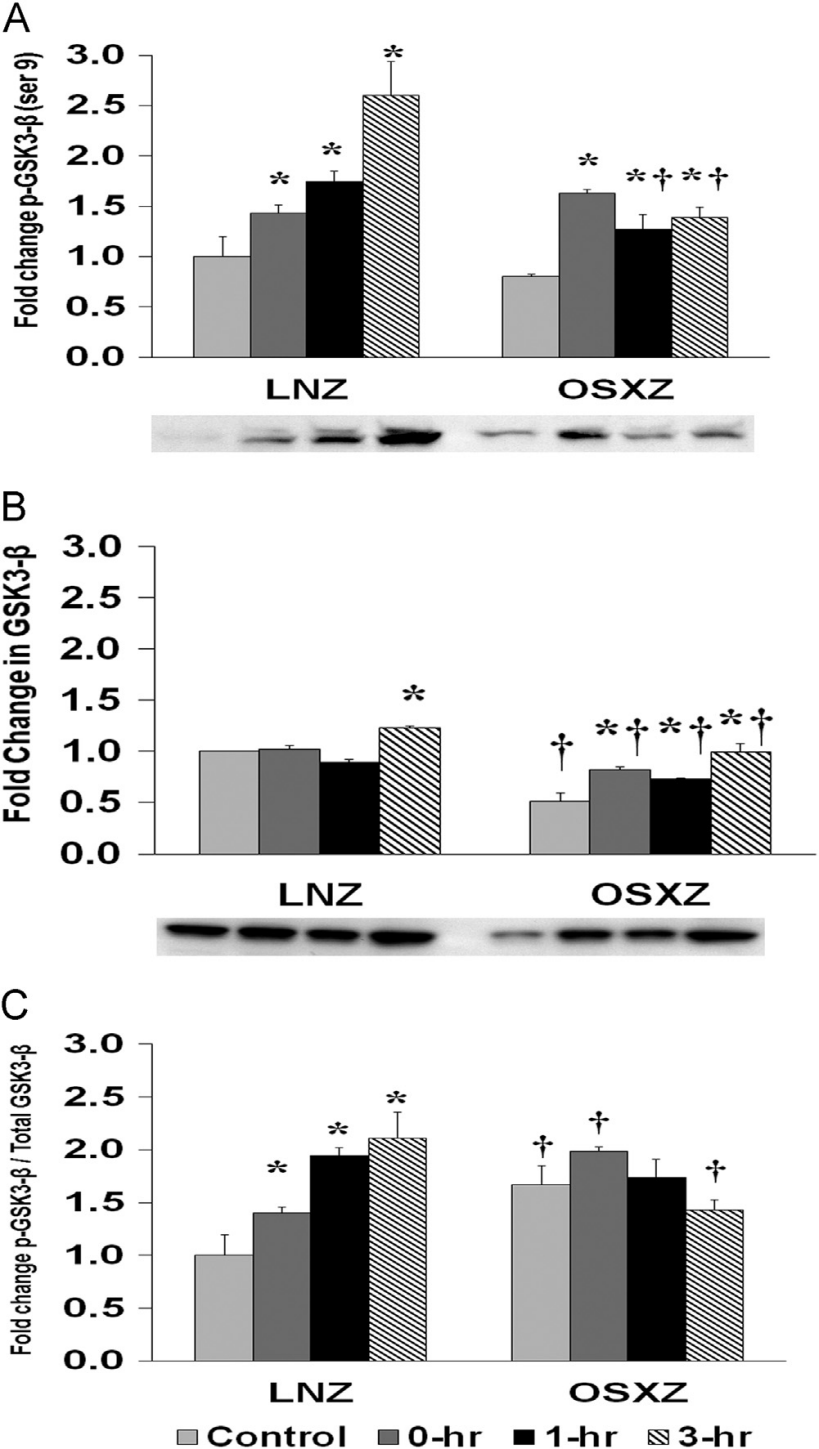
Diabetes alters HFES-induced expression and phosphorylation of GSK3β rat EDL. The basal (control) and HFES-induced expression of GSK3β in EDL from non-diabetic lean Zucker (LNZ) and diabetic obese syndrome X Zucker (OSXZ) rats. * Significantly different from HFES EDL within the same group (*p* < 0.05). † Significantly different from corresponding LNZ EDL (*p* < 0.05). *n* = 6/group.

To determine the effect of HFES on EDL from OSXZ and LNZ animals we evaluated the phosphorylation of GS3K-β at Serine 9. EDL basal phosphorylation of GS3K-β Ser 9 demonstrated no significant difference in the OSXZ when compared to LNZ (Fig. 2A). HFES resulted in an increase (42.9 ± 8.0%, 74.3 ± 10.7%, and 159.9 ± 33.7%, at 0, 1,and 3 h, *p* < 0.05) in phosphorylation of GS3K-β Ser 9 in the LNZ EDL when compared to LNZ contralateral control (Fig. 2A). HFES resulted in an increase (83.0 ± 3.9%, 47.4 ± 14.7%, and 58.9 ± 10.0%, at 0, 1,and 3 hours, *p* < 0.05) in GS3K-β Ser 9 in OSXZ EDL when compared to OSXZ contralateral control (Fig. 2A).

To determine the effect of HFES on EDL from OSXZ and LNZ animals we evaluated the expression of GS3K-β. EDL basal GS3K-β content demonstrated a significant decrease (48.3 ± 8.1%, *p* < 0.05) in the OSXZ when compared to LNZ (Fig. 2B). HFES resulted in a decrease in GS3K-β in the LNZ EDL (23.0 ± 2.1%, at 3 h, *p* < 0.05) when compared to LNZ contralateral control (Fig. 2B). HFES resulted in an increase (30.8 ± 2.3%, 21.3 ± 1.3%, and 47.5 ± 8.5%, at 0, 1,and 3 h, *p* < 0.05) in the OSZX EDL when compared to contralateral OXSZ control (Fig. 2B).

To determine the effect of HFES on EDL from OSXZ and LNZ animals we evaluated the phosphorylation of GS3K-β Ser 9 to total GS3K-β. EDL basal phosphorylation of GS3K-β Ser 9 to total GS3K-β was lower (48.3 ± 8.1%, *p* < 0.05) in the OSXZ when compared to LNZ (Fig. 2C). HFES resulted in an increase in phosphorylation of GS3K-β Ser 9 to total GS3K-β in the LNZ EDL (23.0 ± 2.1% at 3 h, *p* < 0.05) when compared to LNZ contralateral control (Fig. 2C). HFES resulted in an increase in phosphorylation of GS3K-β Ser 9 to total GS3K-β in the OSXZ EDL (30.8 ± 2.3%, 21.3 ± 1.3%, and 47.5 ± 8.5%, at 0, 1 and 3 h, *p* < 0.05) when compared to OSXZ contralateral control (Fig. 2C).

### 1.3 mTor

**Fig. 3:**
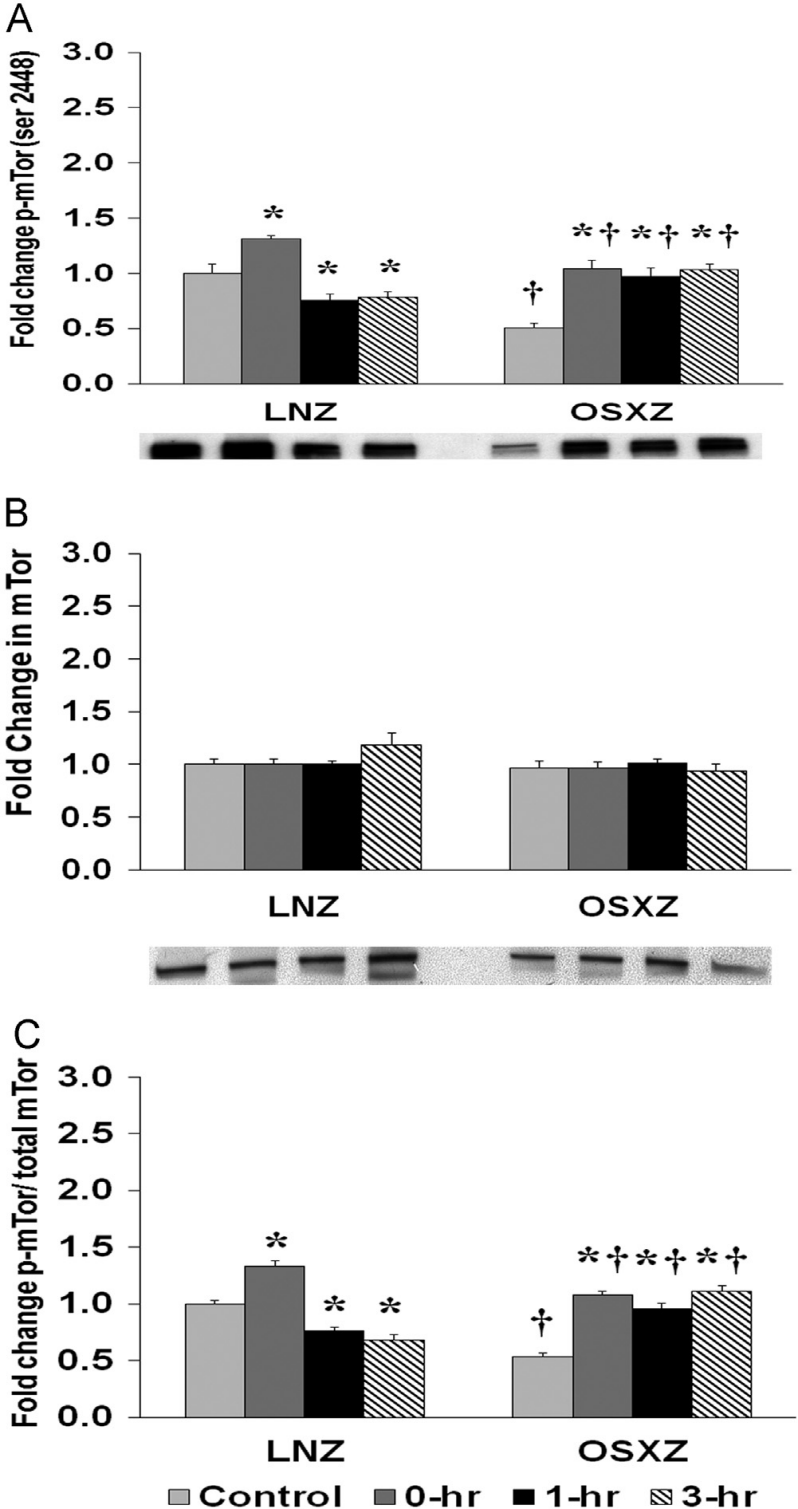
Diabetes alters HFES-induced expression and phosphorylation of mTor rat EDL. The basal (control) and HFES-induced expression of mTor in EDL from non-diabetic lean Zucker (LNZ) and diabetic obese syndrome X Zucker (OSXZ) rats. * Significantly different from HFES EDL within the same group (*p* < 0.05). † Significantly different from corresponding LNZ EDL (*p* < 0.05). *n* = 6/group.

To determine the effect of HFES on EDL from OSXZ and LNZ animals we evaluated the phosphorylation of mTor at Serine 2448. EDL basal phosphorylation of mTor Ser 2448 was lower (49.3 ± 4.3%, *p* < 0.05) in the OSXZ when compared to LNZ (Fig. 3A). HFES resulted in an increase in phosphorylation of mTor at Serine 2448 in the LNZ EDL (31.1 ± 2.7%, at 0 h, *p* < 0.05) and a decrease (24.4 ± 5.3% and 21.7 ± 5.2%, at 1 and 3 h, *p* < 0.05) when compared to LNZ contralateral control (Fig. 3A). HFES resulted in an increase in phosphorylation of mTor at Serine 2448 in the OSXZ EDL (53.3 ± 7.5%, 46.2 ± 7.6%, and 52.5 ± 5.2%, at 0, 1,and 3 h, *p* < 0.05) when compared to OSXZ contralateral control (Fig. 3A).

To determine the effect of HFES on EDL from OSXZ and LNZ animals we evaluated the expression of mTor. EDL basal mTor content demonstrated no significant difference in the OSXZ when compared to LNZ (Fig. 3B). HFES resulted in no significant change in mTor in the LNZ EDL when compared to LNZ contralateral control (Fig. 3B). HFES did not produce a significant change in mTor in the OSXZ EDL when compared to contralateral OXSZ control (Fig. 3B).

To determine the effect of HFES on EDL from diabetic male OSXZ and LNZ animals we evaluated the phosphorylation of mTor at Serine 2448 to total mTor. EDL basal phosphorylation of mTor Ser 2448 to total mTor was lower (47.0 ± 3.3%, *p* < 0.05) in the OSXZ when compared to LNZ (Fig. 3C). HFES resulted in an increase (33.0 ± 5.1%, at 0 h, *p* < 0.05) and decrease (24.3 ± 3.3%, and 31.9 ± 4.9%, at 1, and 3 h, *p* < 0.05) in phosphorylation of mTor Ser 2448 to total mTor in the LNZ EDL when compared to LNZ contralateral control (Fig. 3C). HFES resulted in an increase in phosphorylation of mTor Ser 2448 to total mTor in the OSXZ EDL (54.7 ± 3.4%, 43.1 ± 4.3%, and 58.1 ± 4.7%, at 0, 1, and 3 h, *p* < 0.05) when compared to OSXZ contralateral control (Fig. 3C).

### 1.4 P70s6k

**Fig. 4:**
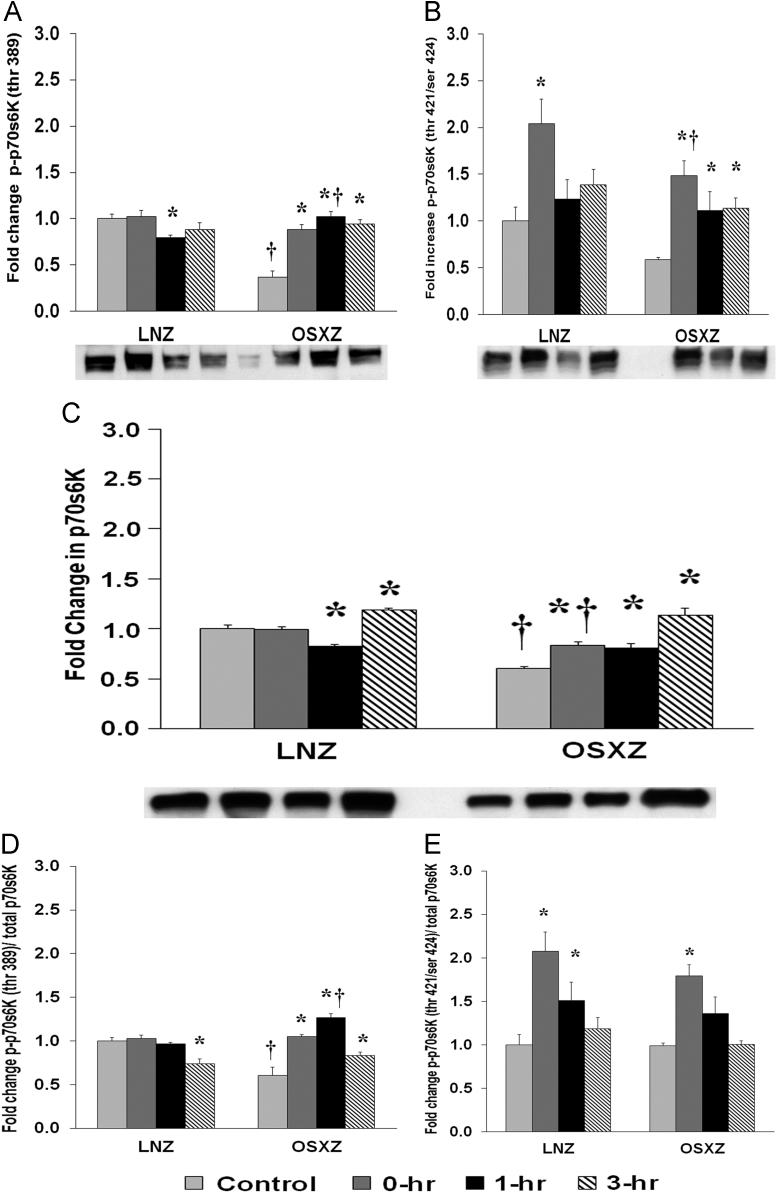
Diabetes alters HFES-induced expression and phosphorylation of p70s6K rat EDL. The basal (control) and HFES-induced expression of p70s6K in EDL from non-diabetic lean Zucker (LNZ) and diabetic obese syndrome X Zucker (OSXZ) rats. * Significantly different from HFES EDL within the same group (*p* < 0.05). † Significantly different from corresponding LNZ EDL (*p* < 0.05). *n* = 6/group.

To determine the effect of HFES on EDL from OSXZ and LNZ animals we evaluated the phosphorylation of p70s6k at Threonine 389 (Thr 389) and Threonine 421 Serine 424 (Thr 421/Ser 424). EDL basal phosphorylation of p70s6k Thr 389 was lower (63.0 ± 6.8%, *p* < 0.05) in the OSXZ when compared to LNZ. HFES resulted in a decrease (20.4 ± 2.6%, at 1 h, *p* < 0.05) in phosphorylation of p70s6k (Thr 389) in the LNZ EDL when compared to LNZ contralateral control (Fig. 4A). HFES resulted in an increase (51.2 ± 5.2%, 64.9 ± 5.9%, and 57.4 ± 4.6%, at 0, 1, and 3 h, *p* < 0.05) in OSXZ EDL when compared to OSXZ contralateral control (Fig. 4A). EDL basal phosphorylation of p70s6k Thr 421/Ser 424 demonstrated no significant difference in the OSXZ when compared to LNZ (Fig. 4B). HFES resulted in an increase (23.4 ± 20.5%, at 0 h, *p* < 0.05) in phosphorylation of p70s6k Thr 421/Ser 424 in the LNZ EDL when compared to LNZ contralateral control (Fig. 4B). HFES resulted in an increase in phosphorylation of p70s6k Thr 421/Ser 424 in the OSXZ EDL (90.0 ± 16.0%, 52.7 ± 20.6%, and 55.0 ± 11.5%, at 0, 1, and 3 h, *p* < 0.05) when compared to OSXZ contralateral control (Fig. 4B).

To determine the effect of HFES on EDL from OSXZ and LNZ animals we evaluated the expression of p70s6k. EDL basal p70s6k was lower (39.9 ± 2.0%, *p* < 0.05) in the OSXZ when compared to LNZ (Fig. 4C). HFES resulted in a decrease (17.9 ± 2.3%, at 1 h, *p* < 0.05) an increase (17.9 ± 2.3%, at 3 h, *p* < 0.05) in p70s6k in the LNZ EDL when compared to LNZ contralateral control (Fig. 4C). HFES resulted in an increase (23.4 ± 3.2%, 20.3 ± .8%, and 53.8 ± 6.9%, at 0, 1 and 3 h, *p* < 0.05) in the OSZX EDL when compared to contralateral OXSZ control (Fig. 4C).

To determine the effect of HFES on EDL from OSXZ and LNZ animals we evaluated the phosphorylation of p70s6k Thr 389 and p70s6k Thr 421/Ser 424 to total p70s6k. EDL basal phosphorylation of p70s6k Thr 389 to total p70s6k was lower (39.6 ± 9.5%, *p* < 0.05) in the OSXZ when compared to LNZ (Fig. 4D). HFES resulted in a decrease in phosphorylation of p70s6k Thr 389 to total p70s6k in the LNZ EDL (26.1 ± 5.8%, at 3 h, *p* < 0.05) when compared to LNZ contralateral control (Fig. 4D). HFES resulted in an increase in phosphorylation of p70s6k Thr 389 to total p70s6k in the OSXZ EDL (44.7 ± 2.3%, 66.6 ± 4.3% and 23.0 ± 3.6%, at 0, 1 and 3 h, p < 0.05) when compared to OSXZ contralateral control (Fig. 4D). EDL basal phosphorylation of p70s6k Thr 421/Ser 424 to total p70s6k was not significant different in the OSXZ when compared to LNZ (Fig. 4E). HFES resulted in an increase (107.8 ± 22.0% and 51.2 ± 21.0%, at 0 and 1 h, *p* < 0.05) in phosphorylation of p70s6k Thr 421/Ser 424 to total p70s6k in the LNZ EDL when compared to LNZ contralateral control (Fig. 4E). HFES resulted in an increase in phosphorylation of p70s6k Thr 421/Ser 424 to total p70s6k in the OSXZ EDL (80.1 ± 13.3%, at 0 h, *p* < 0.05) when compared to OSXZ contralateral control (Fig. 4E).

### 1.5 PTEN

**Fig. 5:**
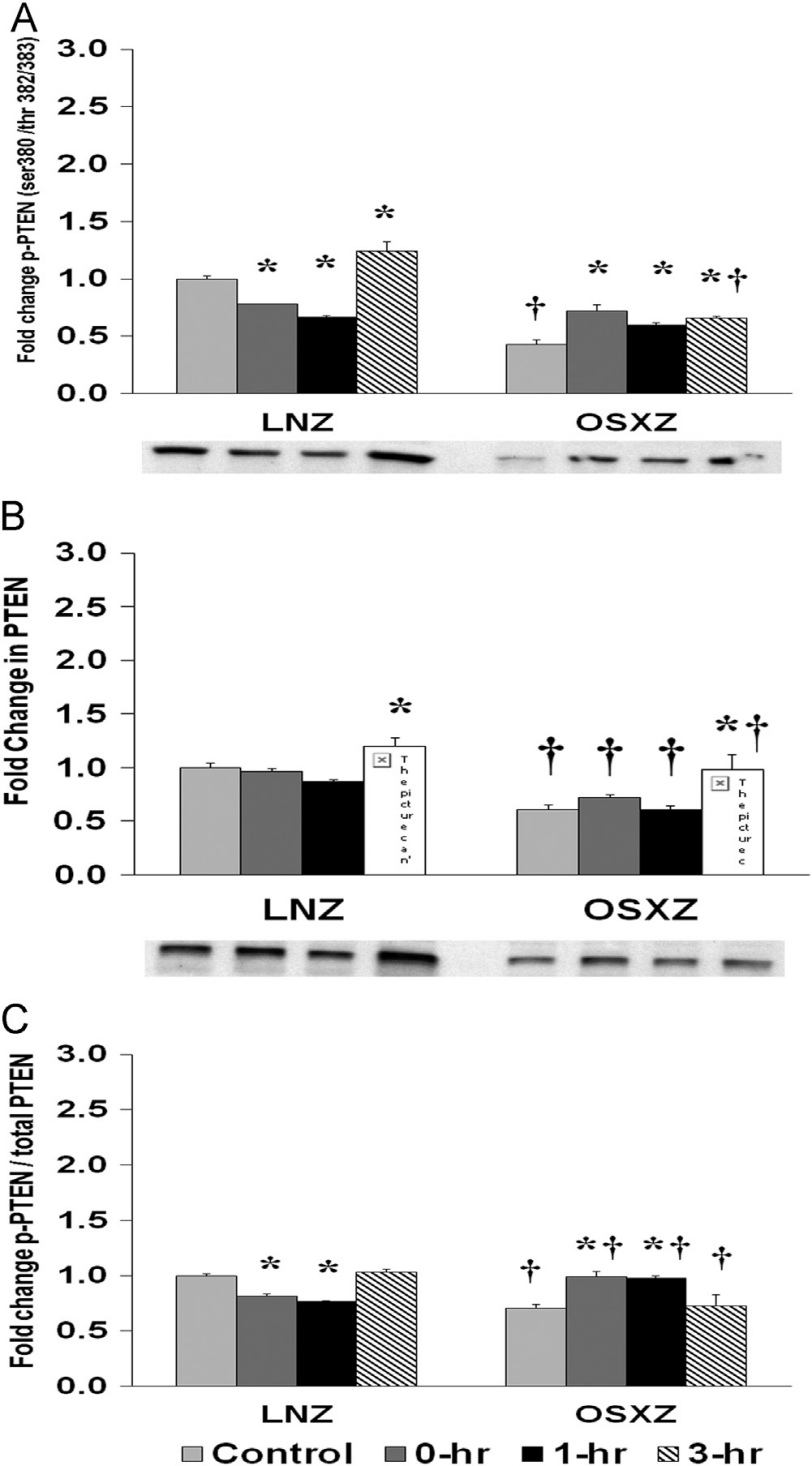
Diabetes alters HFES-induced expression and phosphorylation of PTEN rat EDL. The basal (control) and HFES-induced expression of PTEN in EDL from non-diabetic lean Zucker (LNZ) and diabetic obese syndrome X Zucker (OSXZ) rats. * Significantly different from HFES EDL within the same group (*p* < 0.05). † Significantly different from corresponding LNZ EDL (*p* < 0.05). *n* = 6/group.

To determine the effect of HFES on EDL from OSXZ and LNZ animals we evaluated the phosphorylation of PTEN at Serine 380, Threonine 382 and Threonine 383 (PTEN Ser 380/Thr 382/383). EDL basal phosphorylation of PTEN Ser 380/Thr 382/383 was lower (57.2 ± 4.0%, *p* < 0.05) in the OSXZ when compared to LNZ (Fig. 5A). HFES decreased (22.0 ± 0.3%, and 33.7 ± 1.9%, at 0 and 1 h, *p* < 0.05) and increased (24.5 ± 8.1%, at 3 h, p < 0.05) phosphorylation of PTEN Ser 380/Thr 382/383 in the LNZ EDL when compared to LNZ contralateral control (Fig. 5A). HFES resulted in an increase in phosphorylation of PTEN Ser 380/Thr 382/383 in the OSXZ EDL (29.4 ± 5.2%, 17.0 ± 2.2%, and 22.7 ± 1.4%, at 0, 1, and 3 h, p < 0.05) in OSXZ EDL when compared to OSXZ contralateral control (Fig. 5A).

To determine the effect of HFES on EDL from OSXZ and LNZ animals we evaluated the expression of PTEN. EDL basal PTEN was significant lower (39.4 ± 4.2%, *p* < 0.05) in the OSXZ when compared to LNZ (Fig. 5B). HFES resulted in an increase in PTEN in the LNZ EDL (20.2 ± 7.1%, at 3 h, *p* < 0.05) when compared to LNZ contralateral control (Fig. 5B). HFES resulted in an increase (37.8 ± 13.4%, at 3 h, *p* < 0.05) in PTEN in the OSZX EDL when compared to contralateral OXSZ control (Fig. 5B).

To determine the effect of HFES on EDL from OSXZ and LNZ animals we evaluated the phosphorylation of PTEN Ser 380/Thr 382/383 to total PTEN. EDL basal phosphorylation of PTEN Ser 380/Thr 382/383 to total PTEN was lower (29.7 ± 3.8%, p < 0.05) in the OSXZ when compared to LNZ (Fig. 5C). HFES resulted in a decrease in phosphorylation of PTEN Ser 380/Thr 382/383 to total PTEN in the LNZ EDL (18.6 ± 2.2% and 23.59 ± 0.8%, at 0 and 1 hr, *p* < 0.05) when compared to LNZ contralateral control (Fig. 5C). HFES resulted in an increase in phosphorylation of PTEN Ser 380/Thr 382/383 to total PTEN in the OSXZ EDL (29.0 ± 3.8% and 27.7 ± 4.4%, at 0 and 1 h, *p* < 0.05) when compared to OSXZ contralateral control (Fig. 5C).

### 1.6 SHP2

**Fig. 6:**
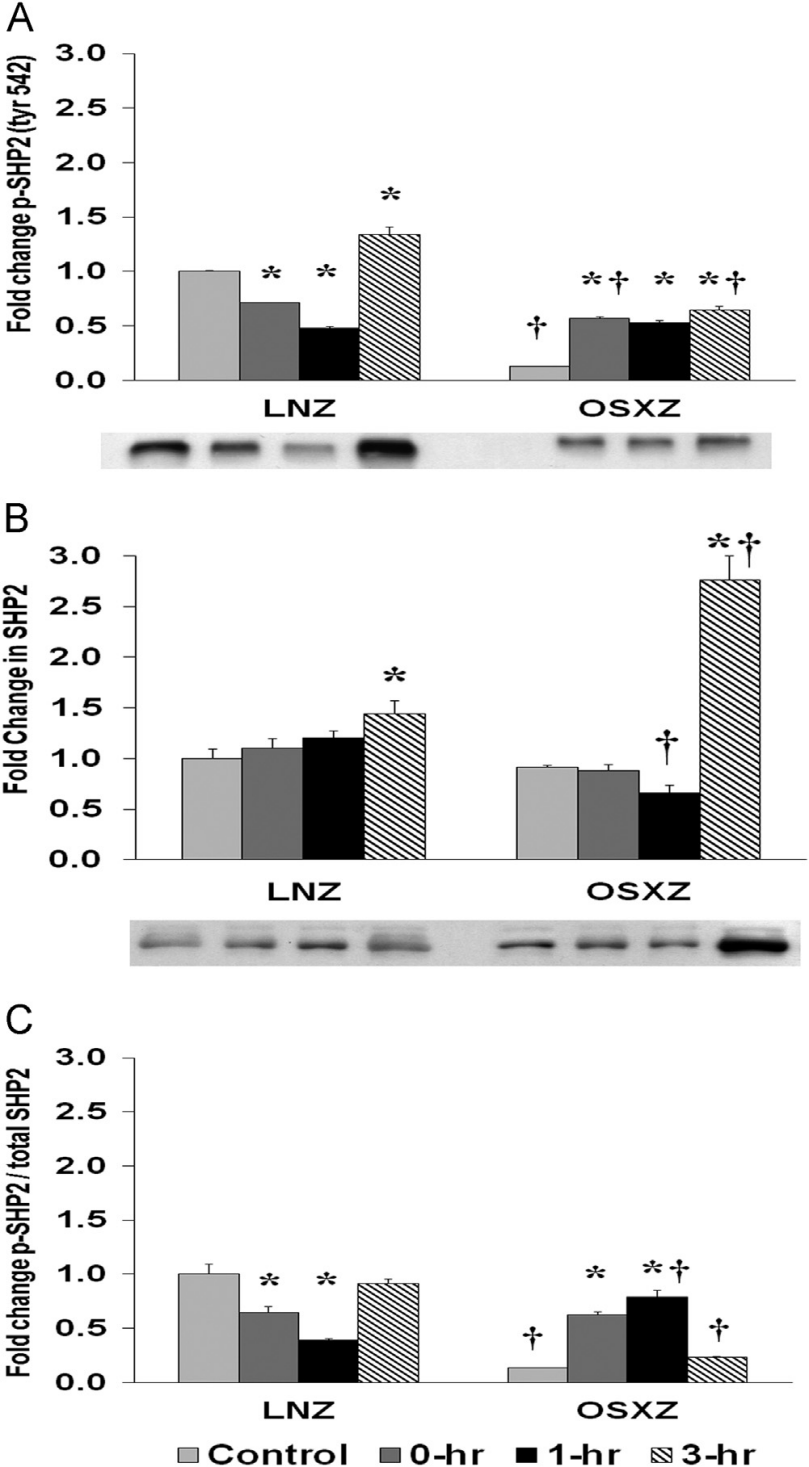
Diabetes alters HFES-induced expression and phosphorylation of SHP2 rat EDL. The basal (control) and HFES-induced expression of p42/p44 in EDL from non-diabetic lean Zucker (LNZ) and diabetic obese syndrome X Zucker (OSXZ) rats. * Significantly different from HFES EDL within the same group (*p* < 0.05). † Significantly different from corresponding LNZ EDL (*p* < 0.05). *n* = 6/group.

To determine the effect of HFES on EDL from OSXZ and LNZ animals we evaluated the phosphorylation of SHP2 at Tyrosine 542. EDL basal phosphorylation of SHP2 Tyr 542 was lower (87.0 ± 0.3%, *p* < 0.05) in the OSXZ when compared to LNZ (Fig. 6A). HFES resulted in a decrease (28.6 ± 0.3% and 51.7 ± 1.0%, at 0 and 1 h, *p* < 0.05) and an increase (33.8 ± 6.8%, at 3 h, *p* < 0.05) in phosphorylation of SHP2 Tyr 542 in the LNZ EDL when compared to LNZ contralateral control (Fig. 6A). HFES resulted in an increase in phosphorylation of SHP2 Tyr 542 in the OSXZ EDL (43.8 ± 1.9%, 39.6 ± 2.3%, and 51.8 ± 3.2%, at 0, 1,and 3 h, *p* < 0.05) when compared to OSXZ contralateral control (Fig. 6A).

To determine the effect of HFES on EDL from OSXZ and LNZ animals we evaluated the expression of SHP23. EDL basal SHP2 content demonstrated no significant difference in the OSXZ when compared to LNZ (Fig. 6B). HFES resulted in an increase in SHP2 in the LNZ EDL (44.0 ± 13.0%, at 3 h, *p* < 0.05) when compared to LNZ contralateral control (Fig. 6B). HFES resulted in an increase (185.1 ± 23.8%, at 3 h, *p* < 0.05) in SHP2 in the OSZX EDL when compared to contralateral OXSZ control (Fig. 6B).

To determine the effect of HFES on EDL from OSXZ and LNZ animals we evaluated the phosphorylation of SHP2 Tyr 542 to total SHP2. EDL basal phosphorylation of SHP2 Tyr 542 to total SHP2 was lower (86.3 ± 0.14%, *p* < 0.05) in the OSXZ when compared to LNZ (Fig. 6C). HFES resulted in a decrease in phosphorylation of SHP2 Tyr 542 to total SHP2 in the LNZ EDL (35.6 ± 5.5% and 61.1 ± 1.4%, at 0 and 1 h, *p* < 0.05) when compared to LNZ contralateral control (Fig. 6C). HFES resulted in an increase in phosphorylation of SHP2 Tyr 542 to total SHP2 in the OSXZ EDL (48.9 ± 2.5% and 65.4 ± 5.7%, at 0 and 3 h, *p* < 0.05) when compared to OSXZ contralateral control (Fig. 6C).

## 2 Experimental design, materials and methods

### 2.1 Animals

All procedures were conducted in strict accordance with the Guide for the Care and Use of Laboratory Animals as approved by the Council of the American Physiological Society and the Animal Use Review Board of Marshall University. Young (10 week, *n* = 12) male lean Zucker (non-diabetic) (LNZ) and young (10 week, *n* = 12) male obese syndrome-X Zucker (diabetic) (OSXZ) rats were obtained from the Charles River Laboratories and barrier housed one per cage in an AAALAC approved vivarium. Housing conditions consisted of a 12H: 12H dark-light cycle and the temperature was maintained at 22 ± 2 °C. Animals were provided food and water *ad libitum*. Rats were allowed to recover from shipment for at least two weeks before the commencement of experimentation during which time the animals were carefully observed and weighed weekly.

### 2.2 Materials

Anti-p70S6k (#9202), Akt (#9272), mTOR (#2972), glycogen synthase kinase-3β (GSK-3β) (#9332), PTEN (#9552), Thr389 (#9206) and Ser 421/Thr 424 (#9204) phosphorylated p70S6K, Thr308 (#9275) and Ser473 (#9271) phosphorylated Akt, Ser2448 phosphorylated mTOR (#2971), Ser 9 phosphorylated GSK-3β (#9336), Ser 380/Thr 382/383 phosphorylated PTEN (#9554), SHP-2 (#3752), p-SHP-2 (Tyr 542) (cat #3751), Mouse IgG, and Rabbit IgG antibodies were purchased from Cell Signaling Technology (Beverly, MA). Enhanced chemiluminescence (ECL) western blotting detection reagent was from Amersham Biosciences (Piscataway, NJ). Precast 10% and 15% SDS-PAGE gels were purchased from Lonza (Rockland, ME). Enhanced chemiluminescence (ECL) western blotting detection reagent was purchased from Amersham Biosciences (Piscataway, NJ). Restore western blot stripping buffer was obtained from Thermo scientific (Rockford, IL) and 3T3 cell lysates from Santa Cruz Biotechnology (Santa Cruz, CA). All other chemicals were from Sigma (St. Louis, MO).

### 2.3 Contractile stimulation of skeletal muscles

The high-frequency electrical stimulation (HFES) model has been previously described [8] and was chosen on the basis of its efficacy in stimulating protein translation and muscle hypertrophy in vivo [9]. The HFES model used in the present study produced 10 sets of 6 contractions with an overall protocol time of 22 min. Animals were killed by a lethal dose of pentobarbital sodium at baseline, immediately following, 1 h or 3 h (*n* = 6 normal, *n* = 6 diabetic for 0, 1, and 3 h) after HFES. Once excised, muscles were blotted dry, trimmed of visible fat and tendon projections, weighed, immediately frozen in liquid nitrogen, and stored at − 80 °C.

### 2.4 Immunoblot analysis

Samples were prepared and immunoblotting conducted as described by Rice et al. [1], [2], [3], [4], [5], [6], [7].

### 2.5 Data analysis

Data were analyzed using Sigma Stat 3.0 statistical software and the results are presented as mean ± SEM. Two-way ANOVA followed by the Student-Newman-Keuls post-hoc testing to determine differences between groups. The level of significance accepted *a priori* was < 0.05.

## References

[1] Katta A., Karkala S.K., Wu M., Meduru S., Desai D.H., Rice K.M., Blough E.R. (2009). Lean and obese Zucker rats exhibit different patterns of p70s6 kinase regulation in the tibialis anterior muscle in response to high-force muscle contraction. Muscle Nerve.

[2] Katta A., Kakarla S., Wu M., Paturi S., Gadde M.K., Arvapalli R., Kolli M., Rice K.M., Blough E.R. (2009). Altered regulation of contraction-induced Akt/mTOR/p70S6k pathway signaling in skeletal muscle of the obese Zucker rat. Exp. Diabetes Res..

[3] Tullgren O., Grimfors G., Holm G., Johansson B., Svedmyr E., Wedelin C., Mellstedt H., Merk K., Bjorkholm M. (1991). Lymphocyte abnormalities predicting a poor prognosis in Hodgkin's disease. A long-term follow-up. Cancer.

[4] Ginjupalli G.K., Rice K.M., Katta A., N.D.P.K M., Arvapalli R., Wu M., Asano S., Blough E.R. (2017). High-frequency electrical stimulation (HFES) Data Lean and Obese Zucker Rat Tibialis Anterior Muscle: regulation of Glycogen synthase kinase 3 beta (GSK3B) Associated Proteins. Data Brief.

[5] Ginjupalli G.K., Rice K.M., Katta A., N.D.P.K M., Arvapalli R., Wu M., Asano S., Blough E.R. (2017). Diabetic Zucker Rat Tibialis Anterior Muscle high-frequency electrical stimulation (HFES) Data: regulation of MAPKs Associated Proteins. Data Brief.

[6] Rice K.M., Katta A., N.D.P.K M., Arvapalli R., Ginjupalli G.K., Wu M., Asano S., Blough E.R. (2017). Lean and Obese Zucker Rat Extensor Digitorum Longus Muscle high-frequency electrical stimulation (HFES) Data: regulation of MAPKs Associated Proteins. Data Brief.

[7] Rice K.M., Katta A., N.D.P.K M., Arvapalli R., Ginjupalli G.K., Wu M., Asano S., Blough E.R. (2017). High-frequency electrical stimulation (HFES) Data Lean and Obese Zucker Rat Soleus Muscle: regulation of p70S6kinase Associated Proteins. Data Brief.

[8] Parkington J.D., Siebert A.P., LeBrasseur N.K., Fielding R.A. (2003). Differential activation of mTOR signaling by contractile activity in skeletal muscle. Am. J. Physiol. Regul. Integr. Comp. Physiol..

[9] Baar K., Esser K. (1999). Phosphorylation of p70(S6k) correlates with increased skeletal muscle mass following resistance exercise. Am. J. Physiol..

